# Identification of methicillin-resistant *Staphylococcus aureus* ST8 isolates in China with potential high virulence

**DOI:** 10.1080/22221751.2022.2031310

**Published:** 2022-02-11

**Authors:** Xinyi Wang, Huilin Zhao, Bingjie Wang, Ying Zhou, Yanlei Xu, Lulin Rao, Wenxiu Ai, Yinjuan Guo, Xiaocui Wu, Jingyi Yu, Longhua Hu, Lizhong Han, Shuying Chen, Liang Chen, Fangyou Yu

**Affiliations:** a Department of Clinical Laboratory, Shanghai Pulmonary Hospital, School of Medicine, Tongji University, Shanghai, People's Republic of China; b Jiangxi Provincial Key Laboratory of Preventive Medicine, School of Public Health, Nanchang University, Nanchang, People’s Republic of China; c Department of Laboratory Medicine, The First Affiliated Hospital of Wenzhou Medical University, Wenzhou, People’s Republic of China; d Department of Jiangxi Provincial Key Laboratory of Medicine, Clinical Laboratory of the Second Affiliated Hospital of Nanchang University, Nanchang, People’s Republic of China; e Department of Laboratory Medicine, Ruijin Hospital, Shanghai Jiao Tong University School of Medicine, Shanghai, People’s Republic of China; f Center for Discovery and Innovation, Hackensack Meridian Health, Nutley, NJ, USA; g Department of Medical Sciences, Hackensack Meridian School of Medicine, Nutley, NJ, USA

**Keywords:** Methicillin-resistant *Staphylococcus aureus*, ST8, SCC*mec* cassette, virulence, pathogenicity

## Abstract

Methicillin-resistant *Staphylococcus aureus* (MRSA) ST8 strains have spread worldwide, causing outbreaks in various regions. However, this clone has only been sporadically reported in China. Consequently, detailed information regarding the phylogeny and potential virulence of *S. aureus* ST8 strains in China remains unknown. In this study, we characterized six ST8 strains collected from three tertiary hospitals in China, including three MRSA (MR50, MR526, and MR254) and three MSSA (H78, H849 and H863). Whole genome sequencing and phylogenetic analysis showed that the six strains formed two separate clusters, including two (MR50 and MR526) and four (MR254, H78, H849 and H863) isolates, respectively. Among them, MR50 and MR526 harboured *spa* t008, SCC*mec* IVa, arginine catabolic mobile element, and were phylogenetically close to the epidemic USA300 strains, while other four strains belonged to *spa* t9101 and formed a unique branch. MR254 carried a novel hybrid SCC*mec* element (namely SCC*mec*254). Same as the USA300 prototype strain LAC, the China *S. aureus* ST8 strains produced weak biofilms except MR254. Among them, MR254 had significantly stronger haemolysis ability and higher α-toxin levels than others, while MR526 showed comparable haemolysis and α-toxin production levels as USA300-LAC. In mouse skin abscess model, MR254 showed particularly strong invasions, accompanied by necrosis, while MR526 exhibited similar infection levels as USA300-LAC. These data suggested that the China MRSA ST8 isolates (e.g. MR254 and MR526) were highly virulent, displaying higher or similar virulence potential as the epidemic USA300 strain. Active surveillance should be enacted to closely monitor the further spread of these hyper-virulent MRSA strains in China.

## Introduction

*Staphylococcus aureus*, one of the major causes of hospital- and community-acquired infections, is an opportunistic pathogen that can cause skin and mucosal infections, as well as endocarditis, osteomyelitis and sepsis [1]. The clinical use of the semi-synthetic antibiotic methicillin led to the first appearance of methicillin-resistant *S. aureus* (MRSA) in 1961, and infections caused by MRSA are more complex and have a higher mortality rate than those caused by methicillin-sensitive strains [2].

Multi-locus sequencing typing (MLST) and SCC*mec* typing have been used to identify and distinguish bacterial isolates as well as clarify the epidemiology of pathogenic bacteria [3,4]. In the United States, the most prevalent sequence type of *S. aureus* are ST8 and ST5 [5–7]. The ST8, SCC*mec* IV (ST8-IV) clone known as USA300 was first identified in the North American and became highly prevalent [8]. USA300 is a super virulent clone, belonging to community-associated MRSA (CA-MRSA) and harbouring SCC*mec* IVa, which can cause severe invasive infections [9,10]. The ST8-IV clone gradually spread around the world; however, the epidemiology of this clone varies significantly in different regions [9]. In Europe, ST8 is a common CA-MRSA, but the most of them are non-USA300 [11]. However, reports of ST8-IV clone (USA300) in Europe are increasing. [12]. In Asia, ST8 is very rare [13].

In China, *S. aureus* clones are mainly ST239, ST59, ST5 and ST398 based on the epidemiological investigations from various regions [14–17]. ST59 was a representative clone of CA-MRSA, showing higher virulence than hospital-associated (HA-MRSA) in the same region [17]. *S. aureus* clones of ST338, ST188, ST45, ST1 and ST7 also emerged and became the common clones [18–20]. *S. aureus* ST8 was rarely isolated from humans in mainland China, although there was a recent report on the emergence of ST8 strains causing bloodstream infections in Taiwan [21]. As reported in previous studies [14,18,22–25], *S. aureus* ST8 has emerged sporadically and distributed in different areas in China mainland. Consequently, there is very limited information on the virulence of *S. aureus* ST8 in China. In addition, the origin of *S. aureus* ST8 is still unclear and their phylogenetic relationship to epidemic USA300 strains remains unknown.

In the present study, we performed whole genome sequencing (WGS) and phylogenetic analysis on six *S. aureus* ST8 isolates emerging in China, and compared them with USA300-LAC, a prototypic *S. aureus* ST8 strain of USA300 from the United States. We then phenotypically and genetically characterized these isolates, and explored their phylogeny, genomic characteristics and virulence potentials.

## Materials and methods

### Ethics statement

This study was approved by the Ethics Committee of Shanghai Pulmonary Hospital, School of Medicine, Tongji University, Shanghai, China. All procedures involving human participants were performed in accordance with the ethical standards. All individual patients or their legal guardians provided informed consent. All animal assays were approved by the Institutional Animal Care and Use Committee of Shanghai Pulmonary Hospital, School of Medicine, Tongji University, Shanghai (Project number: K19-028Y).

### Bacterial isolates

Six clinical isolates of *S. aureus* named MR50, MR526, MR254, H78, H849 and H863 were respectively isolated from three tertiary hospitals in China. The isolates were collected from pus of a 25-year-old woman from Zhejiang Province with perianal abscess in 2020 (MR50), pus of a 69-year-old woman from Zhejiang Province with rectal malignancy in 2019 (MR526), the sputum of a 33-year-old man from Shanghai with pulmonary infection in 2020 (MR254), the blood of a 56-year-old man from Jiangxi Province with renal insufficiency in 2016 (H78), the blood of a 50-year-old man from Jiangxi Province with gout in 2018 (H849) and the blood of a 63-year-old woman from Jiangxi Province with chronic kidney disease in 2020 (H863). The *S. aureus* isolates were cultured and identified in routine microbiology laboratories.

### WGS and analysis

WGS of the six strains was performed by United Medical Technology Co., Ltd., Shenzhen, China using Illumina Hiseq platform. The raw data were filtered by Trimmomatic v0.39 [26], followed by assembly using Spades v3.15.2 [27]. MR254 was also subject to long-reads sequencing using a PacBio Sequel System, and the resulting reads were hybrid assembled with Illumina reads using Unicycler v0.4.9 [28]. The SCC*mec* and *spa* types were determined using SCC*mec*Finder [29] and SpaFinder [30], respectively. The *agr* type was detected by *agr*-group-specific multiplex PCR (detailed information is provided in Supplementary Materials). Antimicrobial resistance genes were examined by AMRFinderPlus v3.10.5 [31]. Virulence gene was mined using blast against the VFDB database (mgc.ac.cn/VFs/), and prophage was determined using PHASTER online tool. Core SNP analysis was conducted following the method described in our recent study [32] using *S. aureus* USA300-FPR3757 genome (accession no. CP000255.1) as the reference. To determine the phylogenetic relationship between China ST8 and the global ST8 genomes, *S. aureus* genome assemblies were downloaded from NCBI RefSeq database (dated as 08/01/2021), and the MLST was determined using mlst v2.19.0 (github.com/tseemann/mlst). The ST8 assemblies were then extracted (*n* = 2173), followed by removing highly similar strains using Assembly-dereplicator (github.com/rrwick/Assembly-Dereplicator) with a threshold of 0.001. The resulting 1087 assemblies and the 6 genomes sequenced in this study were used to generate a core snp phylogenetic tree using ParSNP from Harvest 1.12 [33], followed by visualization and annotation using iTOL v6 [34]. The SCC*mec* in MR254 was compared with other available SCC*mec* elements (SCC*mec* IVa/SCC*mec* V) using Blastn. Easyfig software was used to generate comparison.

### Growth curve assay

The bacteria were cultured in TSB for 12–16 h at 37°C and 220 rpm with shaking. The *S. aureus* strains were then reactivated to a consistent OD_600_ of 0.3 for use. The cultures were transferred into TSB at a ratio of 1:200, with an initial determination of OD_600_. Place all cultures into a shaker at 37°C and 220 rpm. OD_600_ was measured every hour up to 24 h. The growth curve of each strain was repeated three times.

### Antimicrobial susceptibility testing

Antimicrobial susceptibility testing of 11 antimicrobial agents including erythromycin, clindamycin, tetracycline, ciprofloxacin, quinupristin-dalfopristin, cefoxitin, gentamicin, mupirocin, rifampin, linezolid and vancomycin was determined according to the protocols provided by Clinical and Laboratory Standards Institute (CLSI, 2020). *S. aureus* ATCC 29213 and ATCC 25923 strains were used for quality control.

### Biofilm semi-quantitative assay

The *S. aureus* strains were grown in TSB at 37°C overnight with shaking (200 rpm), and the bacteria cultures were diluted 1:100 in TSB containing 1% glucose. The cultures were put into 96-well plate with three replicates per strain and incubated for 24 h. The supernatant was then discarded and sterile phosphate-buffered saline (PBS) was used to wash the wells for three times. About 99% methanol was added and incubated for 15 min to fix biofilms. After discarding the supernatant, 2% crystal violet was added and incubated for 10 min. The stained wells were gently rinsed with water until the water was colorless. The OD value was measured at 600 nm. *S. epidermidis* 12228 and 35984 were used as a negative control and a positive control, respectively. The biofilm-forming capacity was determined according to the following criteria: OD_600_ ≤ Ac (absorbance cut-off value) indicates non biofilm production; Ac < OD_600_ ≤ 2× Ac indicates weak biofilm production; 2× Ac < OD_600_ ≤ 4× Ac indicates moderate biofilm production; OD_600_ > 4× Ac indicates strong biofilm production. The absorbance cut-off value was calculated by OD_600_ of negative control + 3× SD of negative control. The assay was repeated three times in three different days.

### Haemolysin activity determination

The *S. aureus* strains were grown in TSB at 37°C overnight with shaking. After 24 h of incubation, bacteria cultures were normalized to an optical density value 1 at 600 nm (OD_600_). The supernatant was collected by centrifuging at 8000 rpm for 5 min. Then 100 µl of supernatant was added to 900 µl PBS supplemented with 3% sterile defibrillation rabbit blood. We used Triton X-100 as a positive control and PBS as a negative control. After an hour of incubation at 37°C, the absorbance of the supernatant after centrifugation was measured at 600 nm. The assay was performed in triplicate.

### Quantitative enzyme-linked immunosorbent assay and Western blot analysis for α-toxin

The *S. aureus* strains were cultured in TSB for 24 h and adjusted to a same absorbance at OD_600_. The supernatant was collected after centrifuging at 8000 rpm for 5 min, followed by filtering with a 0.22-µm filter. The α-toxin levels were measured by *Staphylococcal* α-Toxin ELISA kit (Chenglin Biological Technology Co., LTD, Beijing, China) following the manufacture’s instructions. Each sample was conducted in triplicate and repeated twice. α-Toxin expression of the *S. aureus* strains was also determined by Western blotting (details in Supplementary Materials).

### Real-time fluorescence quantitative PCR

The *S. aureus* strains were incubated overnight at 37°C with shacking. The total RNA was extracted using a Spin Column Bacteria Total RNA Purification Kit (Sangon Biotech, Shanghai, China) according to the kit instruction. Subsequently, the cDNA was synthesized from purified total RNA using a PrimeScript RT reagent kit (Takara, Japan). Real-time PCRs were performed in a 20-μL system including cDNA, primers and TB Green Premix EX Taq II (Takara, Japan). The expression level of the target gene was calculated by the formula 2^−ΔΔCt^ while *gyrb* was served as the internal reference gene. The primer pairs used in RT–PCR are listed in Table 1. Each reaction was performed in triplicate.

**Table 1.** Primer pairs used in RT-PCR.

Primer	Sequence (5′ → 3′)
*gyrb*-RT-F	ACATTACAGCAGCGTATTAG
*gyrb*-RT-R	CTCATAGTGATAGGAGTCTTCT
*hla*-RT-F	CTCGTTCGTATATTACATCTAT
*hla*-RT-R	GGTATATGGCAATCAACTT
*lukF-PV*-RT-F *lukF-PV*-RT-R	ATTATTACCTATCCAGTGAAGTTG TGCCAGTGTTATCCAGAG
*lukS-PV*-RT-F	CGCTACTTGTATCTTCTGTT
*lukS-PV*-RT-R	CATTGTCGTTAGGAATAATCAC
*saeR*-RT-F	GTCGTAACCATTAACTTCTG
*saeR*-RT-R *mecA*-RT-F	ATCGTGGATGATGAACAA GCGACTTCACATCTATTAG
*mecA*-RT-R	GTGCGATTGTATTGCTAT

### Quantitative enzyme-linked immunosorbent assay for Panton-Valentine Leukocidin

The *S. aureus* strains were cultured in TSB and adjusted to a same absorbance at OD_600_. The supernatant was collected after centrifuging at 8000 rpm for 5 min, followed by filtering with a 0.22-µm filter. The concentration of Panton-Valentine Leukocidin (PVL) production was detected by PVL ELISA kit (Y-J Biological Technology Co., LTD, Shanghai, China) following the manufacture’s instructions. Each sample was conducted in triplicate and repeated twice.

### Mouse skin abscess model

Six-week-old, female BALB/C-nu mice were used for the mouse skin abscess model. The *S. aureus* strains were cultured for 9 h in TSB to the post-exponential phase. After centrifugation at 5000 × g for 10 min, the bacterial cells were washed twice and re-suspended in sterile saline. Mice were randomly divided into four groups with seven mice each group (*n* = 7). Each mouse was injected subcutaneously with 100 μL saline containing 1 × 10^7^ bacterial cells in the buttocks. Mice injected with sterile normal saline were served as the negative controls. After inoculation, the status of abscess in mice were observed for seven consecutive days. The size of abscess area was calculated by the formula of length multiplied by width. A representative nude mouse was selected from each group and skin abscess tissues were dissected for pathological section at 48 h post-infection.

### Statistical analysis and genome accessions

All data were analyzed using GraphPad Prism (version 8.0, La Jolla, CA, United States). Unpaired, two-tailed t-tests were performed to analyze statistical significance and a *p*-value <.05 was considered statistically significant. Error bars in the graphs indicated the standard deviation (mean ± SD). The sequences of MR254 has been uploaded to GenBank (accession no. CP083728-CP083730).

## Result

### *S. aureus* strains and patient history

*S. aureus* MR50 was isolated from perianal pus in the First Affiliated Hospital of Wenzhou Medical University on 28th February 2020, one day after the admission. The patient was admitted to the colorectal anal surgical ward with the diagnosis of perianal abscess and pulmonary nodules. Laboratory tests revealed a slightly elevated leukocyte count (11.43*10^9^/L; normal range: 4–10*10^9^/L), with a neutrophil rate of 74.3%. Cefdinir and fusidic acid were given for anti-infective treatment. She was recovered and discharged after three days of hospitalization.

MR526 was isolated from pus of a rectal malignancy patient in the First Affiliated Hospital of Wenzhou Medical University on 25th September 2019. The patient was admitted to the vascular surgery ward at the end of August 2019, due to intermittent hematochezia for ∼40 days. She was diagnosed with rectal malignancy. The patient had a history of diabetes for 5 years and heart disease for 3 years. Laparoscopic radical resection for rectal cancer and pelvic adhesionolysis were performed successfully on 6th September 2019. She was cured and discharged on 4th October 2019.

MR254 was isolated from sputum in Ruijin Hospital, School of Medicine, Shanghai Jiao Tong University on 29th June 2020. The patient was admitted into critical care medicine ward with Guillain-Barre syndrome on 25th June 2020. He suffered from respiratory failure, pulmonary infection and a catheter-related urinary tract infection. The patient was treated with ceftriaxone sodium, dexamethasone and levofloxacin. The situation was improved and he was discharged on 17th September 2020.

H78 and H849 were isolated from the blood cultures on 2nd March 2016 and 17th March 2018 from two different patients in the same hospital (the Second Affiliated Hospital of Nanchang University) in Central China. Both patients were admitted to the same nephrology ward due to renal insufficiency (H78) and gout (H849), respectively. They were discharged from the hospital with improved conditions. H863 was isolated from the blood culture in the Second Affiliated Hospital of Nanchang University on 31st March 2020. The patient was admitted into nephrology ward with chronic kidney disease and was discharged on 3rd April 2020.

### Growth curve assay

To avoid the amount of bacteria affecting the results of subsequent experiment, we detected the growth of *S. aureus* strains. We found there was almost no difference among these strains at all stages of bacterial growth (Supplementary Figure 1).

### Antimicrobial resistance of ST8 strains

Antimicrobial susceptibility testing showed that MR50, MR526, and MR254 were resistant to cefoxitin while H78, H849, and H863 were susceptible to cefoxitin, phenotypically separated them as MRSA and MSSA, respectively. The minimum inhibitory concentrations (MICs) of cefoxitin for three MRSA isolates were respectively 64 μg/mL (MR50), 32 μg/mL (MR526) and 8 μg/mL (MR254) (breakpoint: ≤4 μg/mL S, ≥8 μg/mL R). The disk diffusion result of cefoxitin for three MRSA isolates were, respectively, 13 mm (MR50), 13 mm (MR526), and 20 mm (MR254) (breakpoint: ≥22 mm S, ≤21 mm R).

The six *S. aureus* isolates were susceptible to tetracycline, quinupristin-dalfopristin, gentamicin, rifampin, linezolid, and vancomycin. Among MRSA isolates, MR254 was resistant to clindamycin while MR50 and MR526 were resistant to ciprofloxacin. For MSSA, H849 was resistant to clindamycin and ciprofloxacin, and H863 was resistant to ciprofloxacin while H78 was susceptible to both two agents.

### Molecular characteristics of ST8 strains

The six clinical *S. aureus* isolates belong to ST8. MR50 and MR526 were identified as *spa* t008 by in silico *spa* typing, while MR254, H78, H849 and H863 belonged to *spa* t9101. MR50 and MR526 harbour SCC*mec* IVa and arginine catabolic mobile element (ACME), while MR254 carried a novel SCC*mec* element without significant matches in the SCC*mec*Finder database. This novel SCC*mec* (namely SCC*mec*254) element was further characterized by complete genome sequencing using PacBio and Illumina sequencing (see below). These isolates carry *agr*-group I like USA300-LAC strain. The molecular characters of these ST8 strains were listed in Table 2.

**Table 2.** Molecular characters of *S. aureus* ST8 strains.

Strain	ST	*spa* type	SCC*mec*	*agr* type	*pvl*	ACME
MR50	8	t008	SCC*mec*IVa	*agr* Ⅰ	+	+
MR526	8	t008	SCC*mec*IVa	*agr* Ⅰ	+	+
MR254	8	t9101	SCC*mec*254	*agr* Ⅰ	−	−
H78	8	t9101	MSSA	*agr* Ⅰ	−	−
H849	8	t9101	MSSA	*agr* Ⅰ	−	−
H863	8	t9101	MSSA	*agr* Ⅰ	−	−
USA300-LAC	8	t008	SCC*mec*IVa	*agr* Ⅰ	+	+

These isolates possess more than 60 virulence genes, including some important regulatory and virulent factors such as *hla*, *saeR/S* and *lukF/S-PV*. Notably, MR50 and MR526 possessed *lukF-PV*, *lukS-PV*, *selk* and *selq*, which were frequently associated with CA-MRSA, whereas these genes were not found in MR254, H78, H849 and H863. MR254, H78, H849, and H863 carried *sdrD*, *sdrE*, and *sed*, but these genes were absent in MR50 and MR526.

### Genetic structure of novel SCC*mec* element in MR254 (SCC*mec*V*-*254)

**Figure 1.**
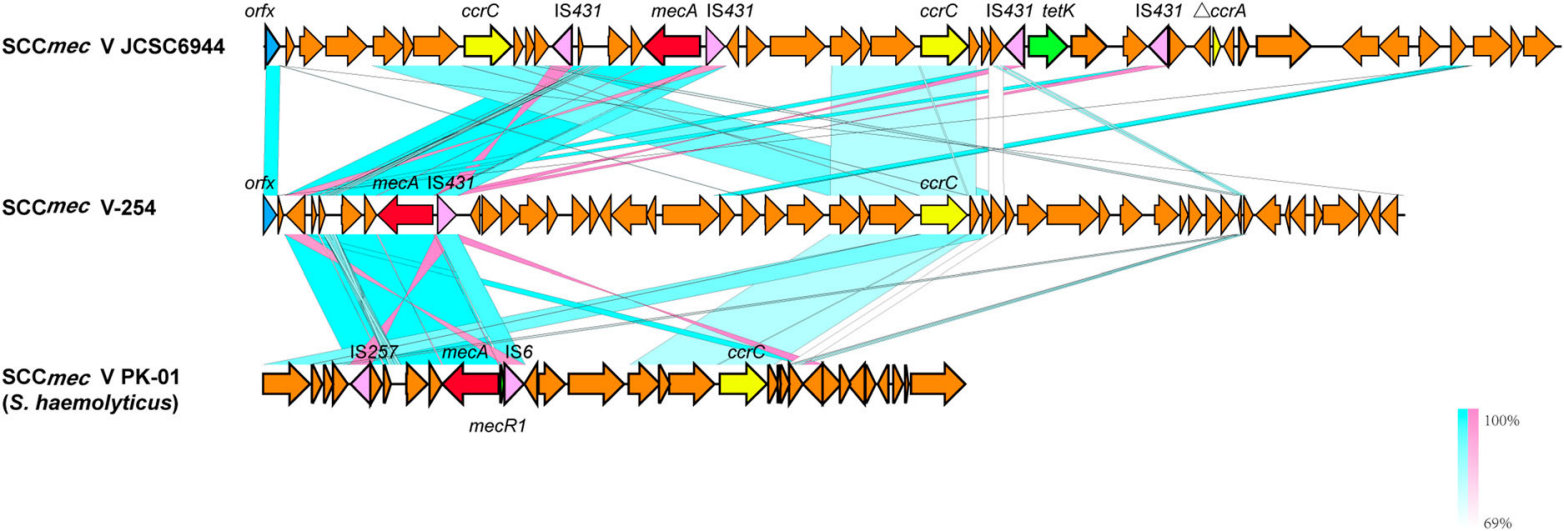
Comparison of SCC*mec* V-254 and two available types of SCC*mec*.

The SCC*mec* region in MR254 (SCC*mec* V-254) was 41.5 kb in length, showing lower than 40% query coverage against all known SCC*mec* elements from the SCC*mec*Finder database. BLAST search against the NCBI nr database showed that SCC*mec*254 had the highest query coverage (69–70%) and identities (98.9–99.3%) against the SCC*mec* region from *S. haemolyticus* (e.g. strain PK-01, accession no. CP035541). It harbours a *mec* class C2 gene complex, which showed 99.98% nucleotide identity to that of SCC*mec* V strain JCSC6944 (AB505629). It also contains a *ccrC* gene; however, it only shows 88.4–94.5% nucleotide identities to the *ccrC*1-10 alleles, and 68.3% to the *ccrC*2 allele, suggesting a novel *ccrC* allele. The joining regions (J regions) of SCC*mec*254 were rather complex and rarely matched to those of other SCC*mec* types (Figure 1). Based on the combination of *mec* complex (*mec* class C2) and *ccrC*, we tentatively name this element SCC*mec* type V variant 254 (SCC*mec* V-254).

### Phylogenetic and comparative genomic analysis

**Figure 2.**
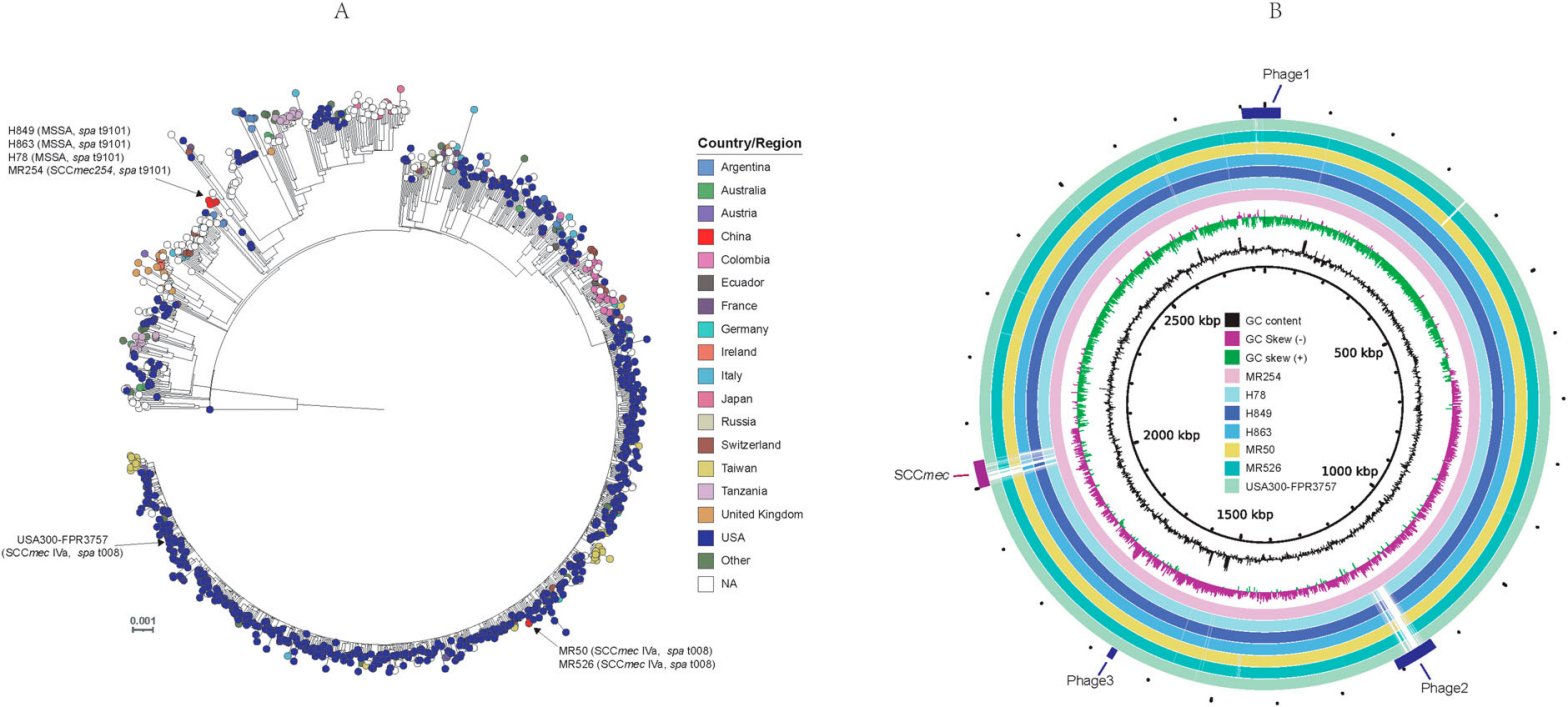
Analysis for the genomes of six *S. aureus* ST8 isolates and USA300- FPR3757. (A) ParSNP phylogenetic tree of 1092 *S. aureus* genomes from RefSeq database and the present study. (B) Comparison of the genomes of MR254 and other *S. aureus* ST8 strains. Each circle represents one strain, MR254, H78, H849, H863, MR50, MR526 and USA300-FPR3757 from the inside out. The SCC*mec* cassette and phage regions are marked in the outermost ring.

The core snp phylogenetic analysis showed that *spa* t9101 strains MR254, H78, H849, and H863, forming a unique clade, separated with the *spa* t008 and SCC*mec* IVa strains MR50 and MR526 (Figure 2(A)). MR50 and MR526 located in the same clade of USA300 isolates, including isolates mostly from USA. Compared with the USA300 prototype strain USA300-FPR3757, MR254, H78, H849, and H863 had an average of 586 core snps (range: 579–590), while MR50 and MR526 had 114 (113–115) core snp difference. MR254, H78, H849, and H863 differ each other with 95 (range: 1–124) core snps, while MR50 and MR526 were only different with 4 core snps. H849 only had one core snp difference with H863, suggesting they were the same strain. Besides the snp variation and the difference of SCC*mec* reigon, MR254 carries three prophages. The phage 3 was shared other ST8 strains, and phage 1 was also found in MR526, H78, H849, and H863. However, the phage 2 was unique to MR254 and was absent in other ST8 strains, including the USA300 reference strain FPR3757 (Figure 2(B)). Based on the phylogenetic and comparative genome analysis results, we selected two MRSA isolates (MR526 and MR254) and one MSSA (H78) for further phenotypic assays and the USA300 strain LAC was included as a reference strain for comparison.

### Biofilm semi-quantitative assay

The biofilm formation levels were compared among the three clinical *S. aureus* ST8 isolates (MR526, MR254, and H78), USA300-LAC and the biofilm producing positive and negative control *S. epidermidis* strains 35984 and 12228. The biofilm formation levels of MR526, MR254, H78, and USA300-LAC were significantly lower than that of the positive control strain 35984 (*p *< .0001) (Supplementary Figure 2). According to the criteria, MR526, H78 and USA300-LAC were weakly positive in biofilm-forming while MR254 was negative.

### Haemolysin activity determination

**Figure 3.**
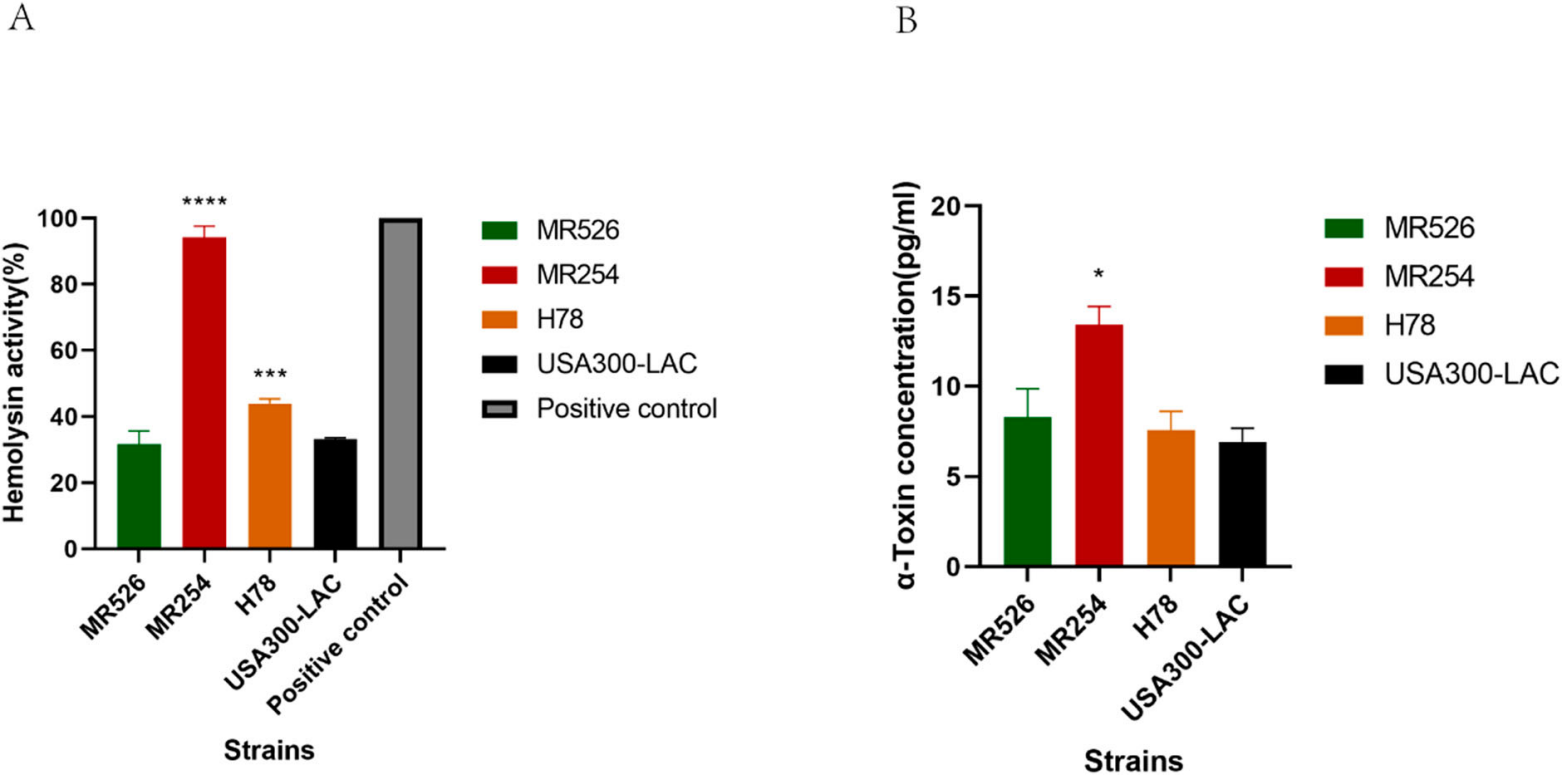
Haemolysis ability of *S. aureus* ST8. (A) Haemolysis phenotype of *S. aureus* ST8 strains. The absorbance measured at 600 nm of each sample was converted to a percentage with A_600_ of the positive control value as 100%. ***p < .001, ****p < .0001. (B) The α-toxin expression of *S. aureus* ST8 strains quantified by ELISA. * represents *p *< .05.

Haemolysis ability is an important determinant of virulence in *S. aureus*. As shown in Figure 3(A), MR526 showed comparable haemolysis ability as that of USA300-LAC (*p *>* *.05); however, H78 and MR254 showed significantly stronger haemolysis abilities (vs. LAC) (*p *< .001), of which MR254 had the highest haemolysis ability (*p *< .0001).

### Quantitative enzyme-linked immunosorbent assay and Western blot analysis for α-toxin

We then quantified the α-toxin production of *S. aureus* ST8 strains using ELISA. The results showed that MR526 and H78 had similar levels of α-toxin production as that of USA300-LAC, while MR254 had significantly higher α-toxin concentration than the other three strains (*p *< .05) (Figure 3(B)). Though Western blotting, α-Toxin production in MR254 was much higher than the other three strains, which was consistent with the ELISA results (Supplementary Figure 3).

### Real-time fluorescence quantitative PCR

**Figure 4.**
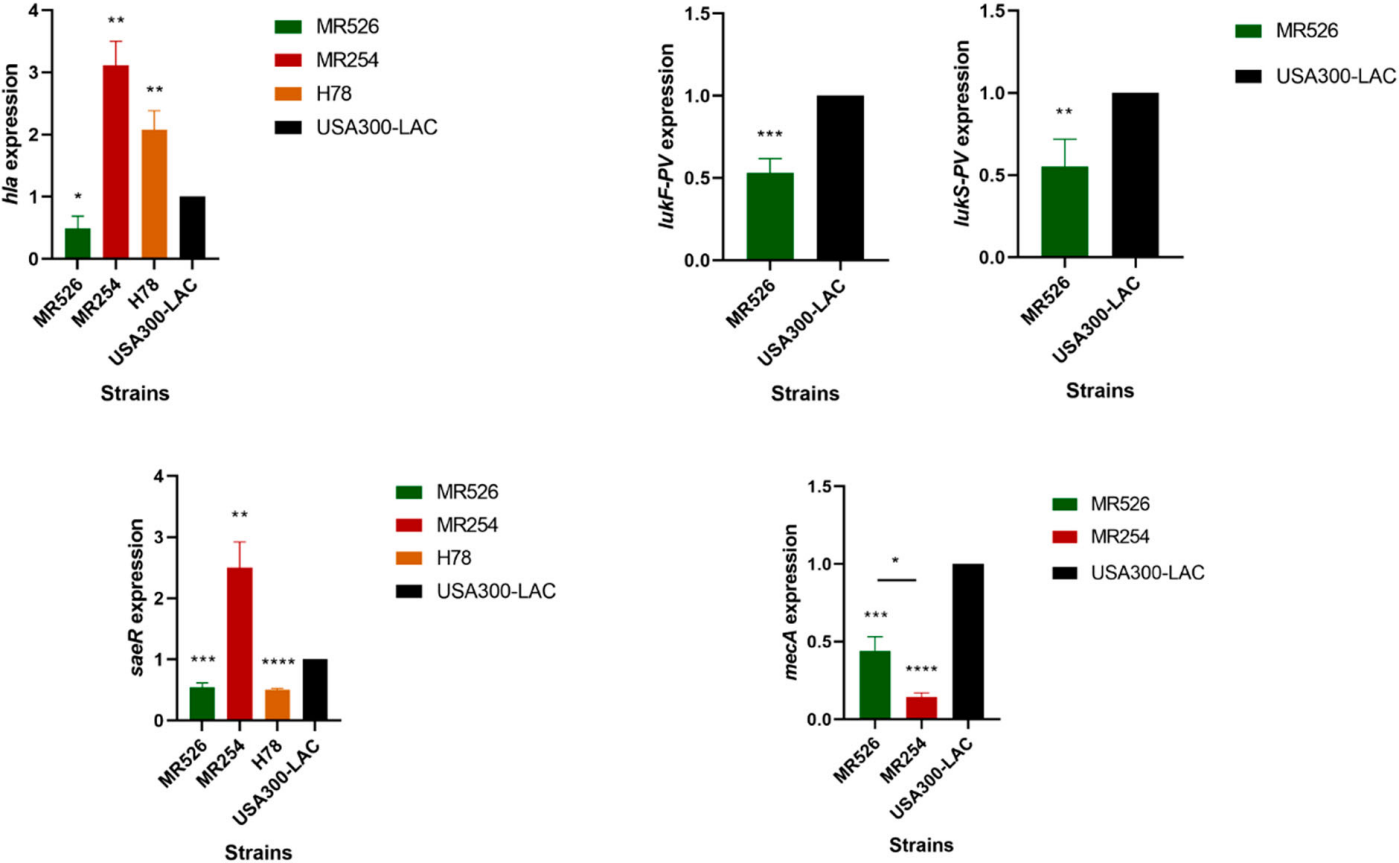
Relative expressions of *hla*, *saeR*, *mecA*, *lukF-PV* and *lukS-PV* in *S. aureus* ST8 isolates. *p < .05, **p < .01, ***p < .001, ****p < .0001.

We firstly performed RT–PCR to investigate the expressions of virulence gene *hla* and *pvl* in *S. aureus* strains (Figure 4). The *hla* expressions of MR254 and H78 were higher while the expression of MR526 was lower than that of USA300-LAC. The expression level of *hla* in MR254 was the highest among all the tested strains, which was consistent with the α-toxin production results. Since MR254 and H78 were negative for *pvl*, only the *lukF-PV/lukS-PV* gene expressions in MR526 and USA300-LAC were examined. We found that the expression levels of *lukF-PV* and *lukS-PV* in MR526 was lower than that in USA300-LAC. The expression of regulatory gene, *saeR,* was also examined. The levels of *saeR* expression in MR254 were significantly higher but the expressions in MR526 and H78 were lower than that of USA300-LAC. Moreover, we also examined the relative expressions of *mecA* gene in MRSA strains MR526, MR254, and USA300-LAC. As shown in Figure 4, the *mecA* expressions of MR526 and MR254 were lower than that of USA300-LAC, and the level of MR254 was the lowest (*p *< .05).

### Quantitative enzyme-linked immunosorbent assay for Panton-Valentine Leukocidin

**Figure 5.**
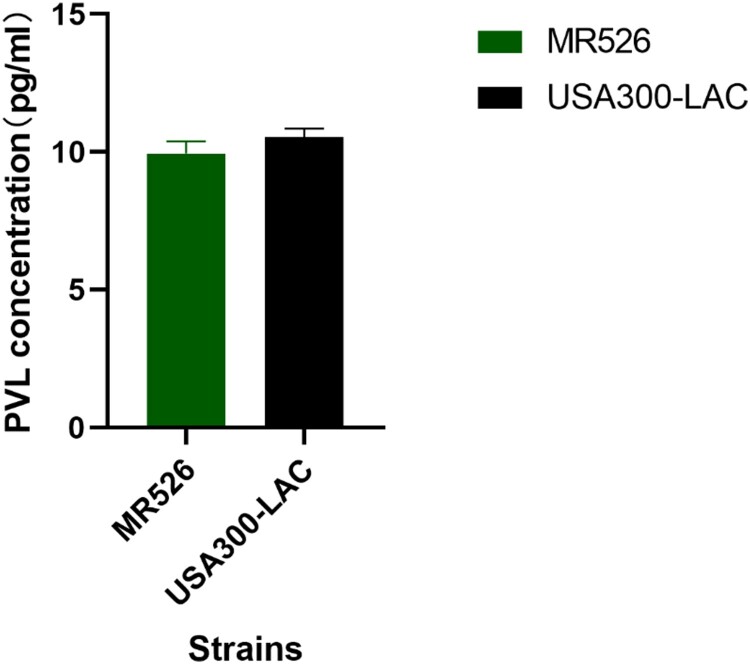
The PVL expression of *S. aureus* ST8 strains.

We also measured the concentration of PVL in the supernatant of bacteria cultures using ELISA. We found that MR526 exhibited lower levels of PVL production than USA300-LAC as shown in Figure 5.

### Virulence comparison of the ST8 isolates in a mouse skin abscess model

**Figure 6.**
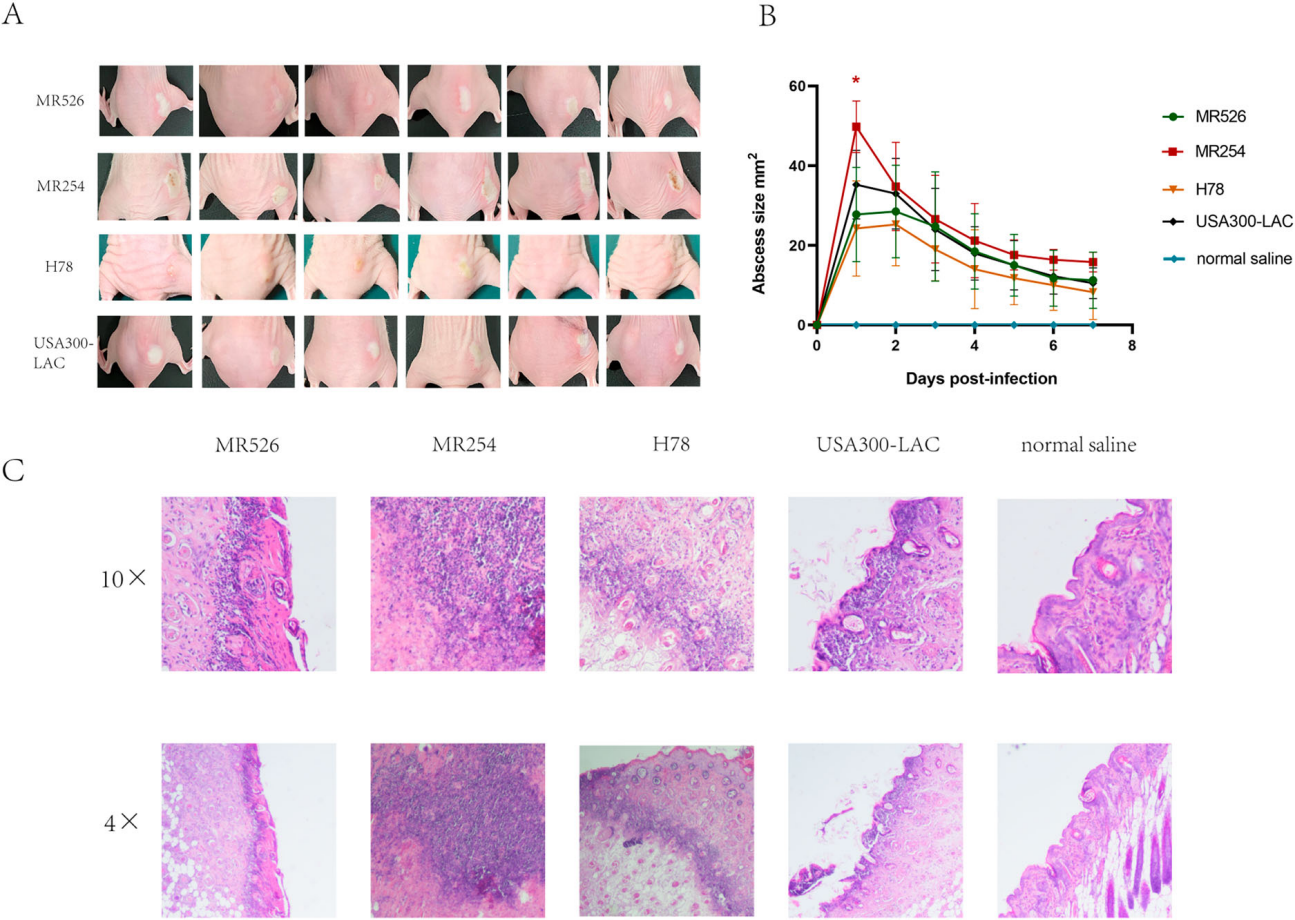
The mouse skin infections caused by *S. aureus* ST8. (A) Abscesses caused by *S. aureus* ST8. The lesion sizes of six mice in each group on the first day post-infection were shown. (B) The abscess size changes after infections pattern. (C) Pathological sections of the representative mice from each group, including two power fields (4× and 10×) of skin abscess tissues dissected.

We then employed a murine skin abscesses model to evaluate the in vivo virulence of the ST8 strains causing skin and soft tissue infections (SSTIs). The four strains showed variable ability to cause skin abscesses in mice (Figure 6(A)). MR526 caused more severe abscesses than H78, and the abscesses sizes were equivalent to those of USA300-LAC. Notably, the abscesses caused by MR254 were more serious than all the other tested strains, including USA300-LAC, and the abscess sizes in MR254 group were significantly larger (49.80 mm^2^ vs. 35.25 mm^2^ in LAC on the first day post-infection, *p *< .05). The changes in abscess size of mice were plotted to Figure 6(B). The abscesses were most pronounced on the first and second day after infection and then started to reduce and gradually heal. We found that MR526 and H78 caused less severe abscesses than USA300-LAC while MR254 caused more significant ones. These findings were consistent with *saeR* expression levels of four *S. aureus* ST8 strains as the previous study suggested the virulence in animal skin infection models was associated with the expression of *SaeR* [35]. Pathological sections showed that the four ST8 strains caused neutrophil infiltration in the epidermis layer (Figure 6(C)). Degrees of neutrophil infiltration in MR526 and USA300-LAC were similar while the neutrophil infiltration in H78 was minimal. Notably, neutrophil infiltration induced by MR254 was obviously strongest, with accompanied necrosis. These findings suggested that two MRSA ST8 isolates had strong potential to cause skin infections, especially MR254, which was highly virulent with the ability to invade the skin.

In order to further observe the virulence level of MRSA ST8 MR254, we selected invasive MRSA strains of ST59 and ST398 to establish a comparison with MR254. As shown in Supplementary Figure 4, the abscess sizes in MR254 group were significantly larger (58.15 mm^2^ vs. 32.10 mm^2^ in ST59-MR40 on the first day post-infection, *p* < .05). And the abscess sizes in ST59-MR40 group were larger than ST398-MR173. The skin lesions infected by MR254 were more serious than ST59-MR40 and ST398-MR173. Pathological sections showed that MR254 caused the most severe neutrophilic infiltration, accompanied with extensive necrosis. ST59-MR40 caused neutrophil infiltration with partial necrosis which was lighter than MR254. ST398-MR173 resulted in only neutrophil infiltration without necrosis. These findings can be supportive of the high virulence of MR254.

## Discussion

The ST8-IV clone (USA300) was first recognized as a cause of skin and soft tissue infection in 2000 in the USA [36]. However, USA300 isolates also caused more serious infections including bacteraemia, endocarditis, severe necrotizing pneumonia, and osteomyelitis [36]. The USA300 clone had spread globally and gradually became a synonym for severe community-associated staphylococcal disease [37]. This epidemiologically successful clone pertains to t008 *spa* type and usually carries characteristic SCC*mec* IVa element, ACME and *pvl* genes [9].

Compared with America, where the USA300 clone predominates [12], *S. aureus* ST8 isolates has only been sporadically described in various regions of China, including a MSSA isolate collected in infected neonate from Guangxi Province [18], a MRSA isolate collected in nasal of a 17-year-old *S. aureus* carrier from Guangzhou Province [22] and a CA-MRSA isolated from pus of a 39-year-old patient in Sichuan Province [23]. However, most of those previous reports have focused on molecular epidemical description, while detailed phenotypic and genomic characterizations of China ST8 strains remain to be limited. In most cases, the authors only mentioned ST8 strains, without detailed infection descriptions. Among ST8 isolates reported before, three caused bloodstream infection, two caused skin and soft tissue infection, one caused respiratory tract infection and one colonized in humans without causing disease. In particular, genomic and phylogenetic analysis of China ST8 strains is lacking. To the best of our knowledge, this is the first comprehensive study, phenotypically and phylogenetically determining the China ST8 isolates.

In our study, six *S. aureus* ST8 clinical isolates were collected from different regions in China. Among them, MR50 and MR526 harboured the same genomic signatures as the epidemic USA300 strains, including *spa* t008, SCC*mec* IVa, PVL and ACME. Phylogenetic analysis also placed them in the same cluster as those USA300 strains from the US, suggesting the two strains belonged to the epidemic CA-MRSA USA300 clone. Notably, phenotypic tests (e.g. haemolysin activity and α-toxin quantitation) and mouse skin abscess model demonstrated that MR526 had similar virulence as the prototype USA300-LAC strains, suggesting the China ST8 USA300-like strains (e.g. MR526) did not attenuate virulence after the introduction into a new geographical region. The further spread of these USA300-like strains in the communities and hospitals in China should be closely monitored.

Another MRSA strain, MR254, was located on a separate phylogenetic clade as MR50 and MR526. Additionally, MR254 was PVL-negative and belonged to *spa* t9101. So far, only two *spa* t9101 strains have been submitted to Ridom SpaServer (spaserver.ridom.de), collected from Germany and China, respectively, and both were MSSA. The China *spa* t9101 strain was isolated in 2009, prior to the isolation time of the Germany *spa* t9101 isolate (2012). Several studies have reported the identification of *S. aureus* ST8-t9101 isolates in different regions in China, including an isolate from a SSTI patient in Jiangsu Province (year 2014–2015) [24], an isolate recovered from breast milk in Shanghai Province (year 2015–2016) [25] and two isolates from patients with bloodstream infection (year 2014–2015) [38], indicating that the ST8-t9101 isolates have been circulating in China for over 10 years. Moreover, most of isolates were MSSA. MR254 represents one of the first ST8-t9101 MRSA isolates. The SCC*mec* element in MR254 was distinct with the known SCC*mec* structures identified in the MRSA isolates. Relatively, this element appears to be close to SCC*mec* V. SCC*mec*254 harbours a truncated *mecRI* (only 20 bp residue), a novel *ccrC* and complex J regions. *mecRI* is a regulatory element which can control the expression of the *mec* gene (e.g. *mecA*) through encoding a signal transducer protein; *ccrC* encodes the site-specific recombinases and is phylogenetically different from *ccrAB* (with DNA sequence similarity of less than 50%); and J regions are non-essential but may contain determinants for additional antimicrobial resistance [2].

In our study, three MSSA ST8 isolates (H78, H849, and H863) were also identified as t9101. They were closely related to the MRSA *spa* t9101 strain MR254. We reasoned that the *spa* t9101 MRSA (i.e. MR254) may be evolved from MSSA ST8-t9101 through the acquisition of novel SCC*mec*254 element from other non-*S. aureus* species (e.g. *S. haemolyticus*). Current molecular epidemiology of *S. aureus* in China showed that the spread of ST8-t9101 strains was still sporadic, partly because most of these strains were MSSA. However, the MRSA ST8-t9101 strain appears to be more resistant than the MSSA strains, which may further promote the dissemination of these strains if antibiotic selection pressure is present. Nevertheless, the susceptibility testing showed that MRSA ST8-t9101 strain MR254 has relative lower cefoxitin resistance than the MRSA ST8-IVa-t008 strains MR50 and MR526, and the *mecA* gene expression results also showed that this strain has lower *mecA* expression levels in comparison to MR526. SCC*mec*254 structure analysis showed that it harbours a truncated *mecR1*, and the deficiency of *mecR1* may in turn down-regulate the expression of *mecA* [2]. Consequently, it remains elusive how much the presence of SCC*mec*254 will impact the clinical occurrence of MRSA ST8-t9101 strains in China.

Interestingly, the MRSA ST8-t9101 strain MR254 demonstrated significantly higher virulence potential than the USA300-like strains. In this study, it showed the highest level of haemolysin activity, a-toxin production and abscesses formation. Since it is negative for *pvl*, and therefore, its overall virulence is independent on PVL. The *SaeRS* two-component system is a major virulence regulatory system of *S. aureus* which drives the expression of several important virulence factors, including coagulase, alpha-toxin, β- and γ-haemolysins, Panton-Valentine leucocidin (LukGH), TSST-1, exfoliative toxin and nuclease [35]. Deletion of *saeR/S* down-regulated expression of virulence genes [39,40]. It has been proved that deletion of *saeR/S* in MW2 significantly alters expression of genes encoding *hla*, *hlgA*, *hlgB*, *hlgC*, *sbi*, and adhesins such as fibrinogen-binding proteins [41]. We accordingly suspected that the high expression level of *saeR* in MR254 may play an important role in the process of exerting high virulence by up-regulating other virulence factors. In our present study, it is likely that the conspicuous *hla* expression in MR254 was associated with its high *saeR* expression level. Additional virulence factors regulated by *saeR* may be further discovered. Moreover, α-toxin has been reported to have a link with SSTI through promoting epithelial injury [42]. We speculated that the high level of α-toxin production in MR254 may attribute to serious infections in the mouse skin abscess model. The molecular mechanisms underlying increased *SaeRS* and *hla* expressions still need further studies. Notably, MRSA ST8-t9101 strain MR254 also demonstrated significantly higher virulence than the MSSA ST8-t9101 strain H849. Comparative genomic analysis identified 124 core snp variations between the two genomes. In addition, MR254 contains additional two genomic island regions, including the SCC*mec*254 and a 65-kb prophage (nt 1141194-1207170). Future studies are warranted to examine if the acquisition of the two region or small chromosomal variations correlate with the increased virulence in MR254.

Our study has several limitations. First, the sample size was small, and only six ST8 isolates were included. Although the prevalence of ST8 strains is low in China, sporadic ST8 isolates have been described in different regions in China. Future large-scale and multiregional studies should be conducted to assess the geographical dissemination and clinical impact of ST8 strains in China. Second, bacterial virulence is a complex trait dependent upon numerous attributes including both host and microbe factors. Although our study showed that MR254 and MR526 had higher or comparable virulence as USA300-LAC in the mouse skin abscess model, additional clinically revelent infection models, such as bacteraemia, sepsis, peritonitis and endocarditis, should be considered to assess their overall virulence of causing invasive infections. Last, the genetic factors underlying the hyper-virulence in our ST8 isolates were not explored, and further studies are needed to address their molecular mechanisms.

Taken together, in this study, we phenotypically and genetically characterized the emerging *S. aureus* ST8 strains in China and dissected their phylogenic relationship. Two phylogenetic distinct MRSA ST8 clones were revealed, including one closely related to the epidemic USA300 strains and a new ST8-t9101 strain harbouring a novel SCC*mec*254 element. Notably, both the USA300-like and ST8-t9101 MRSA strains in China showed similar or even higher virulence levels than the prototype USA300-LAC strain, representing an emerging threat to the hospitals and the communities in China. The clinical practice should be alerted about the spread of these highly virulent strains, and active molecular surveillance should be enacted.

## Supplementary Material

Supplemental Material
