# Supplementary material for: Identification of methicillin-resistant Staphylococcus aureus ST8 isolates in China with potential high virulence
Source: Emerg Microbes Infect. 2022 Feb 11;11(1):507–18. doi: 10.1080/22221751.2022.2031310 (PMC8843119; doi:10.1080/22221751.2022.2031310)
Supplement: Supplemental Material [file TEMI_A_2031310_SM7397.docx]

***Agr* group-specific multiplex PCR**

The *S. aureus* strains were incubated overnight at 37℃ with shacking. Genomic DNA was extracted from staphylococci using a Ezup Column Bacteria Genomic DNA Purification Kit (Sangon Biotech, Shanghai, China) according to the kit instruction. The *agr* sequences were amplified in a 25-μl reaction mixture containing the purified nucleic acid, primers and 2× Rapid Taq Master Mix (Vazyme Biotech Co., LTD, Nanjing, China). The primers used in multiplex PCR are as follows: Pan (5′-ATG CAC ATG GTG CAC ATG C-3′), *agr*Ⅰ (5′-GTC ACA AGT ACT ATA AGC TGC GAT-3′), *agr*Ⅱ (5′-TAT TAC TAA TTG AAA AGT GGC CAT AGC-3′), *agr*Ⅲ (5′-GTA ATG TAA TAG CTT GTA TAA TAA TAC CCA G-3′), and *agr*Ⅳ (5′-CGA TAA TGC CGT AAT ACC CG-3′), which allows the amplification of a 441-bp DNA fragment (*agr*-group Ⅰ), a 575-bp DNA fragment (*agr*-group Ⅱ), a 323-bp DNA fragment (*agr*-group Ⅲ), and a 659-bp DNA fragment (*agr*-group Ⅳ), respectively. Multiplex PCR products were analyzed by electrophoresis in 1.5% agarose gels.

**Western blot analysis for α-toxin**

The *S. aureus* strains were cultured in TSB for 24 h and bacterial culture supernatants were collected and washed twice with sterile PBS. The protein concentration of each sample was measured in order to keep the total amount of protein loaded for SDS-PAGE consistent in the four strains. Proteins were denatured at 100 °C for 5-10 min in Omni-Easy^TM^ Protein Sample Loading Buffer (EpiZyme Biotechnology Co., LTD, Shanghai, China). The samples were separated by 10% sodium dodecyl sulfate polyacrylamide gel electrophoresis (SDS-PAGE) and the proteins were blotted onto a polyvinylidene difluoride (PVDF) membrane. After 1 h of blocking at room temperature with 1× PBST buffer containing 5% BSA, the membrane was incubated at 4℃ overnight with Anti-*Staphylococcal* α-Toxin antibody produced in rabbit (Sigma-Aldrich) at a dilution of 1:20000. The membrane was then incubated with Goat Anti-Rabbit IgG HRP (Biosharp) at a 1:5000 dilution ratio. The protein was visualized by Omni-ECL^TM^ Pico Light Chemiluminescence Kit (EpiZyme Biotechnology Co., LTD, Shanghai, China).

**
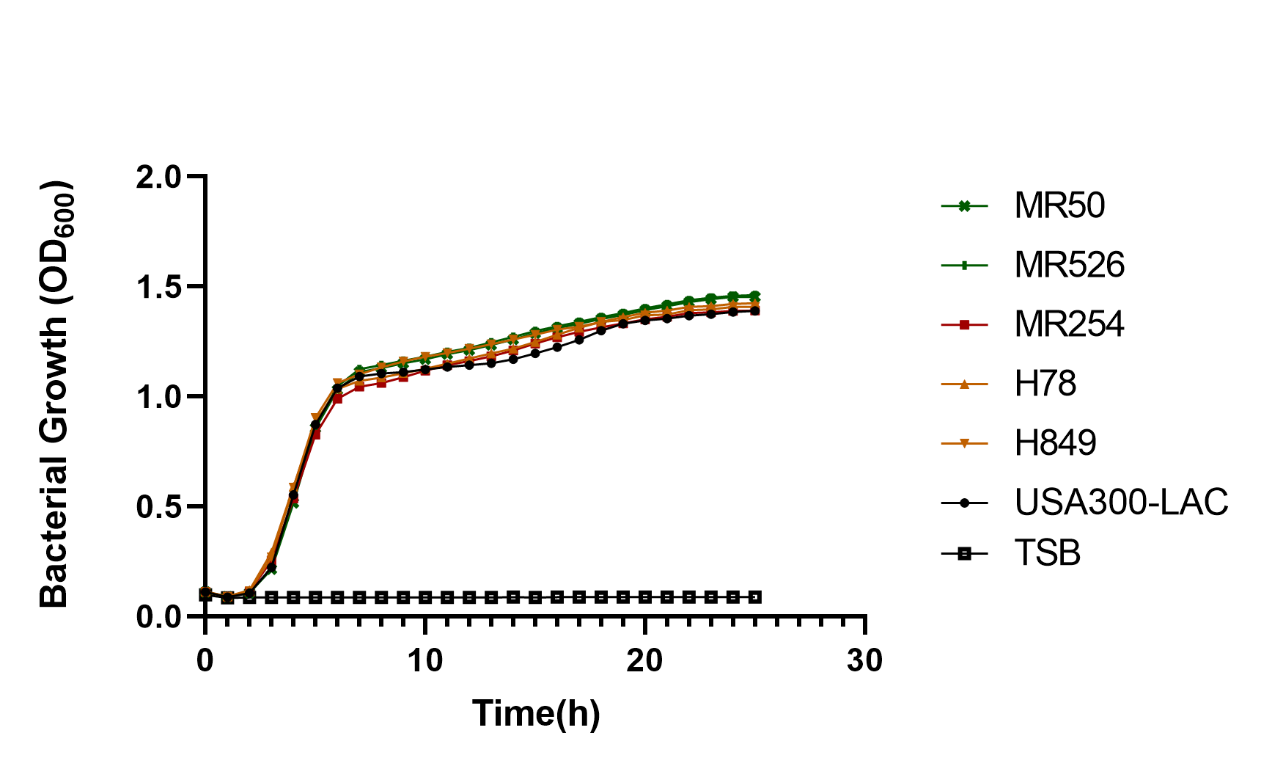
**

**Figure S1** Growth curves of *S. aureus* ST8 strains.

**
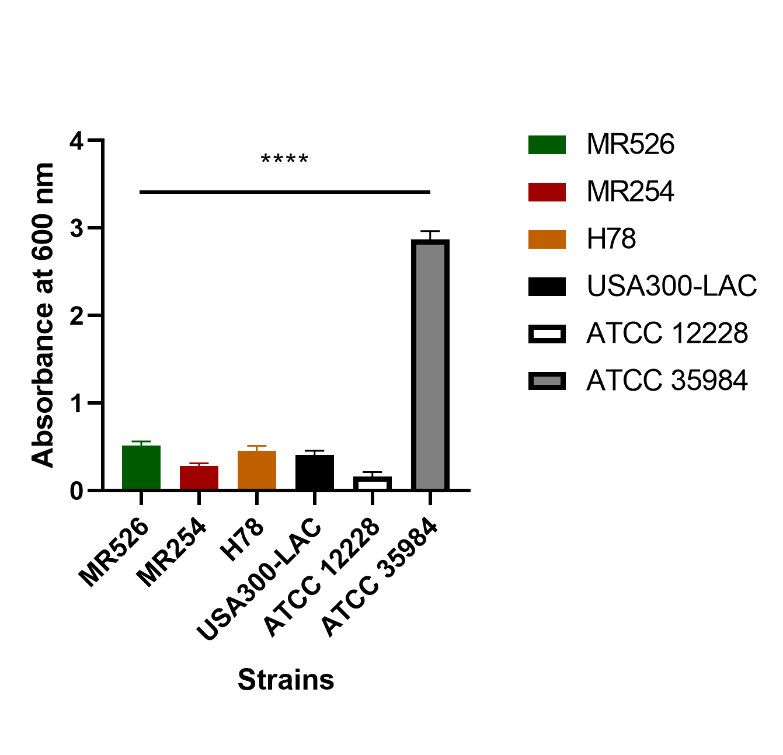
**

**Figure S2** The absorbance measured at 600 nm in biofilm formation assay of *S. aureus* ST8. **** *p*<0.0001.


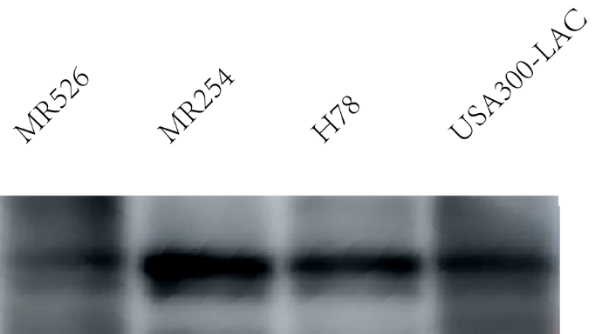


**Figure S3** The α-toxin expression of *S aureus* ST8 strains determined by Western blotting, keeping the total amount of protein loaded for SDS-PAGE consistent in the four strains.

**
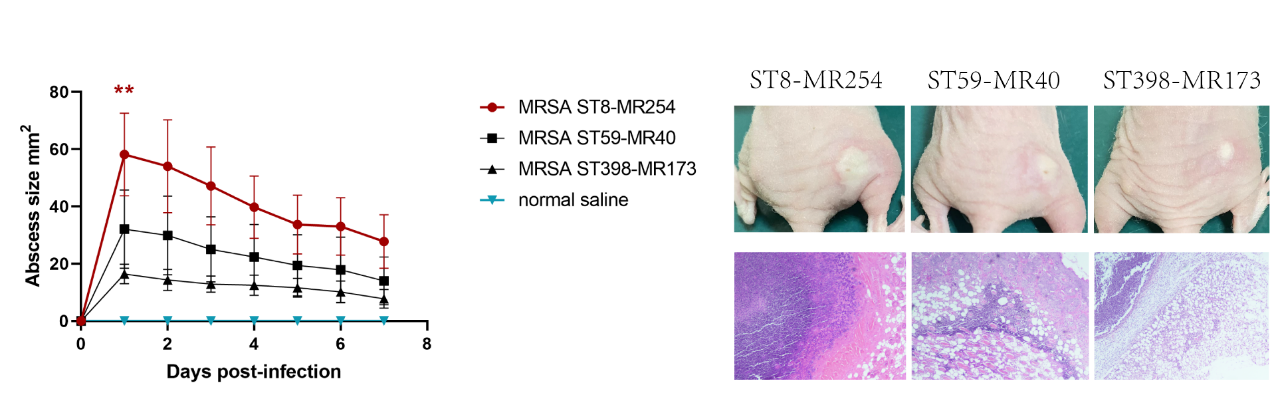
**

**Figure S4** Results of mouse skin infection model experiments. Comparison of abscess sizes, skin lesions and pathological sections (4×) were shown. ** p<0.01.
